# Predicting nanoparticle tumor delivery efficiency using explainable machine learning for cancer nanomedicine design

**DOI:** 10.3389/fbioe.2026.1919132

**Published:** 2026-10-20

**Authors:** Abdul Karim, Sutikno, Arry Maulana Syarif, In cheol Jeong

**Affiliations:** 1 Cerebrovascular Disease Research Center, Hallym University, Chuncheon, Gangwon, Republic of Korea; 2 Department of Artificial Intelligence Convergence, Hallym University, Chuncheon, Gangwon, Republic of Korea; 3 Department of Informatics, Faculty of Science and Mathematics, Diponegoro University, Semarang, Indonesia; 4 Faculty of Computer Science, Universitas Dian Nuswantoro, Semarang, Indonesia; 5 Department of Population Health Science and Policy, Icahn School of Medicine at Mount Sinai, New York, NY, United States

**Keywords:** cancer nanomedicine, explainable artificial intelligence, machine learning, nanomedicine design, nanoparticle drug delivery, shap, tumor biodistribution, XGBoost

## Abstract

Nanoparticle-based drug delivery systems have been proposed as a promising strategy for improving the efficacy and specificity of cancer therapeutics. However, predicting tumor delivery efficiency remains challenging because of complex interactions among nanoparticle physicochemical properties, biological environments, and experimental conditions. This study presents an explainable machine-learning framework for predicting nanoparticle tumor delivery efficiency using the updated Nano-Tumor Database. Following data preprocessing and feature engineering, 2,319 records were used to develop and evaluate Random Forest, Extreme Gradient Boosting (XGBoost), Light Gradient Boosting Machine (LightGBM), weighted ensemble, and stacking ensemble models. To reduce information leakage from repeated nanoparticle measurements, model development and evaluation were revised using nanoparticle-identity-aware grouping based on NM.ID, with categorical preprocessing fitted exclusively on training data. In the final untouched NM.ID-grouped hold-out set, the stacking ensemble achieved the lowest RMSE (3.8107) and highest $R^{2}$ (0.0901), while the weighted ensemble achieved the lowest MAE (1.8320); however, differences among the leading models were small. Five-fold NM.ID-grouped cross-validation yielded mean $R^{2}$ values of 0.2280 for XGBoost, 0.2169 for Random Forest, and 0.1980 for LightGBM, demonstrating that generalization to previously unseen nanoparticle identities is substantially more challenging than suggested by row-wise validation. Repeated group-aware splits further showed variability in model ranking, while stricter reference-grouped validation indicated limited cross-study transportability. SHapley Additive exPlanations (SHAP) were used to examine model interpretability, with Animal, Tumor Cell, Core material, Dose, and Zeta Potential showing the largest global contributions to predictions from the final tuned XGBoost model. Dose showed no meaningful marginal correlation with tumor %ID, although group-aware ablation indicated modest multivariable predictive contribution. Performance generally deteriorated for core-material categories absent from training, indicating that the framework should primarily be interpreted as an explainable screening and hypothesis-generation tool within the represented chemical and experimental domain rather than as a validated predictor for completely novel nanoparticle chemistries. These findings highlight both the potential and current generalization limitations of data-driven approaches for supporting nanoparticle design in cancer nanomedicine.

## 1 Introduction

Despite substantial advancements in diagnosis, targeted therapeutics, immunotherapy, and precision medicine, cancer continues to be a prevalent source of morbidity and mortality globally. A primary difficulty in cancer treatment is the efficient delivery of therapeutic medicines to tumor tissues while minimising systemic toxicity and off-target effects. Nanoparticle-based drug delivery systems are suggested as an effective approach to increase the pharmacokinetic and pharmacodynamic characteristics of anticancer therapies through improved tumor targeting, extended circulation duration, regulated drug release, and diminished side effects. Various nanocarrier platforms, including gold nanoparticles, liposomes, polymeric nanoparticles, lipid nanoparticles, polymeric systems, and hybrid nanostructures, have demonstrated significant promise to improve treatment efficacy across multiple cancer types (Hossain et al., 2024; Ma et al., 2024; Rashidi et al., 2024).

However, the clinical use of cancer nanomedicine is impeded by the insufficient efficacy of nanoparticles in targeting tumor tissues. The biodistribution and accumulation of nanoparticles in tumors are influenced by various biological obstacles, including blood circulation dynamics, immune clearance, vascular heterogeneity, characteristics of the cancer microenvironment, and the physicochemical properties of the nanoparticles (Chen et al., 2023; Lin et al., 2022). Prior extensive assessments have shown that a limited percentage of supplied nanoparticles arrive at tumor locations, highlighting the necessity for systematic strategies to enhance nanoparticle design and delivery efficacy (Chen et al., 2023). Consequently, comprehending the intricate interactions among nanoparticle characteristics, administration variables, and biological elements is essential for the systematic development of advanced nanomedicines.

The conventional experimental optimisation of nanoparticle formulations is frequently time-consuming, costly, and resource-intensive. The growing accessibility of extensive nanomedicine datasets has enabled data-driven methodologies to reveal concealed patterns and associations that may not be easily discernible using conventional experimental techniques (Wang et al., 2025). Recently, machine learning (ML) and artificial intelligence (AI) techniques have demonstrated considerable potential to expedite the advancement of nanomedicines by facilitating the optimization of nanoparticle formulations, forecasting drug release, modelling biodistribution, and predicting therapeutic outcomes (Wang et al., 2025; Santana et al., 2020; Harrison et al., 2021). These computational methods facilitate the integration of several physicochemical and biological characteristics to create predictive models that can be employed in the design of nanoparticles prior to costly experimental validations.

Recently, numerous studies have been undertaken to examine the application of machine learning approaches for predicting nanoparticle delivery. Lin et al. utilized the Nano-Tumor Database to construct machine learning and deep learning models, demonstrating that deep neural networks surpassed conventional approaches in forecasting nanoparticle delivery effectiveness across several cancer models (Lin et al., 2022). Chen et al. subsequently enhanced the Nano-Tumor Database by an extensive meta-analysis of nanoparticle dispersion in tumors and key organs, providing one of the most complete resources accessible for nanomedicine research (Chen et al., 2023). In a recent work, Mi et al. employed various machine learning models to forecast the tissue distribution and tumor delivery efficacy of nanoparticles in mice. They attained commendable prediction performance using deep learning methodologies and identified nanoparticle core material as a pivotal factor affecting delivery efficiency (Mi et al., 2024). Mahdi et al. employed machine learning models alongside feature selection and hyperparameter optimization techniques to forecast the biodistribution of nanoparticles, underscoring the efficacy of data-driven approaches in elucidating nanoparticle behaviour within biological systems (Mahdi et al., 2025a).

These studies revealed the feasibility of employing machine learning to forecast the biodistribution of nanoparticles; yet, numerous hurdles persist. The interpretability of models is crucial, as numerous effective machine learning algorithms function as black-box systems, constraining biological understanding and practical design recommendations. Secondly, comparative assessments of various machine learning and ensemble learning methodologies are necessary utilizing new large-scale nanoparticle datasets. Third, it remains crucial to ascertain the most significant nanoparticle characteristics that affect tumor delivery efficiency, to inform the systematic design of nanocarriers and facilitate clinical translation (Wang et al., 2025; Mi et al., 2024; Chen et al., 2023).

Recent artificial-intelligence approaches have further expanded the computational analysis of nanoparticle delivery beyond conventional tabular prediction. Tang et al. developed a deep generative adversarial network (GANDA) for generating intratumoral nanoparticle distribution patterns, illustrating the potential of deep learning for spatial characterization of nanoparticle delivery (Tang et al., 2021). More recently, Tang et al. proposed an interpretable radiomics-based machine-learning model using routine medical imaging to predict nanomedicine tumor accumulation, demonstrating the growing role of interpretable artificial intelligence in nanoparticle delivery assessment and patient-stratification research (Tang et al., 2025).

The present study builds on, but differs methodologically from, earlier machine-learning analyses of the Nano-Tumor Database. Lin et al. developed machine-learning and deep-learning models using the earlier version of the database, which contained 376 nanoparticle datasets collected from studies published through 2018 (Lin et al., 2022). The subsequently updated Nano-Tumor Database expanded the resource to 534 tumor datasets comprising 2,345 time-dependent tumor concentration data points collected from 297 studies published between 2005 and 2021 (Chen et al., 2023). Thus, the present analysis is based on the expanded database rather than the earlier 376-dataset resource. Ma et al. subsequently proposed an interpretable XGBoost-SHAP framework that integrated nanoparticle physicochemical properties with tumor genomic mutation information to predict selected delivery-efficiency endpoints (Ma et al., 2023). These previous studies establish the value of both machine learning and explainable modelling for nanoparticle delivery prediction, but they also motivate evaluation under validation schemes that explicitly account for repeated nanoparticle identities and heterogeneous source studies.

Accordingly, the principal contribution of the present work is not the introduction of XGBoost or SHAP individually, but a leakage-aware and uncertainty-conscious evaluation of explainable machine learning on the updated Nano-Tumor Database. The framework compares Random Forest, XGBoost, LightGBM, weighted ensemble, and stacking ensemble approaches while explicitly grouping records by nanoparticle identity (NM.ID) during hold-out testing and cross-validation. Hyperparameter optimization and ensemble construction are restricted to training data, and repeated group-aware partitions are used to quantify variability in generalization performance. A stricter reference-grouped sensitivity analysis further evaluates transportability across literature sources. In addition, the study examines the relationship between administered dose and tumor %ID through correlation and group-aware ablation analyses and directly evaluates model behaviour for core-material categories absent from the training partition. SHapley Additive exPlanations (SHAP) are used to characterize influential physicochemical, biological, and experimental predictors. Collectively, these analyses provide a more conservative assessment of predictive generalization and clarify the conditions under which explainable machine-learning models may support nanoparticle screening, while explicitly identifying limitations in extrapolation to new nanoparticle identities, independent studies, and previously unseen material classes.

## 2 Materials and methods

### 2.1 Data source and dataset description

This study used the updated Nano-Tumor Database as the primary data source for developing an explainable machine learning framework to predict nanoparticle tumor delivery efficiency. The database contains experimentally reported nanoparticle biodistribution measurements together with physicochemical properties, tumor model information, animal model characteristics, administration conditions, and time-dependent delivery outcomes. The original Excel file contained 2,645 rows and 21 columns. After removing the descriptive header row and performing data cleaning, the dataset was converted into a structured modeling table.

The prediction target was tumor biodistribution efficiency, reported as percentage injected dose (%ID). The final modeling dataset contained 2,319 complete records after preprocessing and missing-value removal. Ten predictor variables were used for model development, including nanoparticle material, morphology, particle size, surface charge, dose, tumor size, tumor cell type, animal model, polydispersity index, and time point. Table 1 summarizes the final variables used in the machine learning pipeline.

**TABLE 1:** Summary of variables used for nanoparticle tumor delivery prediction.

Variable	Type	Description
Core material	Categorical	Main nanoparticle material composition
Shape	Categorical	Morphological structure of the nanoparticle
Tumor cell	Categorical	Tumor cell or cancer model used in the experiment
Animal	Categorical	Animal model used for biodistribution assessment
Size_num	Numerical	Extracted nanoparticle size from reported measurements
TumorSize_num	Numerical	Extracted tumor size or tumor volume information
Dose_num	Numerical	Extracted nanoparticle administration dose
PDI	Numerical	Polydispersity index of the nanoparticle formulation
Zeta potential	Numerical	Surface charge of the nanoparticle formulation
Time point (h)	Numerical	Biodistribution measurement time point in hours
Biodistribution (%ID)	Target	Tumor delivery efficiency measured as percentage injected dose

### 2.2 Proposed workflow

The workflow of the proposed explainable machine learning system is depicted in Figure 1. The pipeline had seven principal stages: data collecting, data preprocessing, feature engineering, model training, performance evaluation, explainability analysis utilizing SHAP, and prediction output. The methodology was crafted to guarantee transparency, reproducibility, and biological interpretability of the predictive modelling process.

**FIGURE 1:**
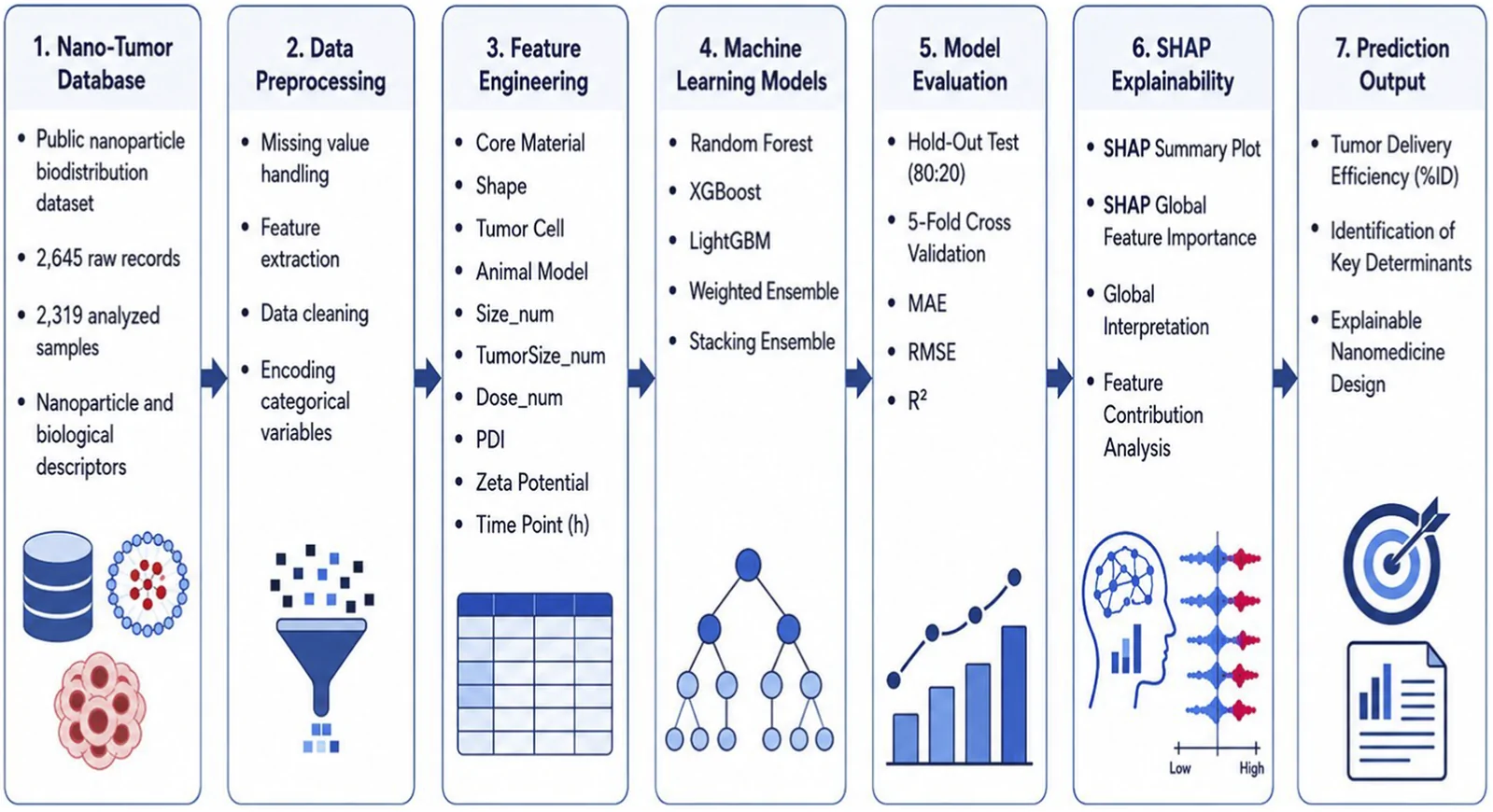
Overall workflow of the proposed explainable machine learning framework for predicting nanoparticle tumor delivery efficiency. The pipeline includes data acquisition from the updated Nano-Tumor Database, preprocessing, feature engineering, model training, evaluation, and SHAP-based explainability analysis.

### 2.3 Data preprocessing

Data preprocessing was conducted to transform the raw database into a machine-readable format suitable for regression modelling. The organ-label information in the initial row of the dataset was eliminated prior to model development. Numerous nanoparticle formulations were documented over various time intervals; hence, repetitive formulation descriptors were propagated to preserve the linkage between each biodistribution data and its respective nanoparticle formulation. Entries with absent values in the target variable and in the final modelling variables were eliminated.

Categorical variables, including core material, nanoparticle shape, tumor cell type, and animal model, were treated as categorical predictors and subsequently transformed using one-hot encoding within the training pipeline. Numerical features, including particle size, tumor size, dose, PDI, zeta potential, and time point, were retained as continuous variables. Text-based fields containing numerical information, such as particle size and administration dose, were parsed to obtain quantitative values. These preprocessing procedures followed standard machine-learning practices while ensuring that categorical transformations were learned exclusively from training data (Bishop, 2006; Goodfellow et al., 2016).

The final modeling dataset comprised 2,319 complete records after preprocessing. Because the database contains repeated measurements associated with the same nanoparticle entries, a conventional row-wise random split can place measurements from the same nanoparticle formulation in both the training and evaluation sets and thereby produce overly optimistic estimates of generalization. To address this issue, all revised model development and primary evaluation procedures were performed using the nanoparticle identifier (NM.ID) as the grouping variable. Thus, records sharing the same NM.ID were kept entirely within either the training or evaluation partition.

For the primary hold-out evaluation, a group-aware 80:20 split was generated using GroupShuffleSplit with a fixed random seed of 42. This resulted in 1,872 training records representing 385 unique nanoparticle identifiers and 447 test records representing 97 unique nanoparticle identifiers, with zero NM.ID overlap between the two partitions. The test partition was kept untouched during preprocessing, hyperparameter selection, ensemble construction, and model development, and was used only for the final evaluation.

Categorical predictors (core material, shape, tumor cell type, and animal model) were encoded using one-hot encoding. The encoder was fitted exclusively to the corresponding training data and configured to permit previously unseen categorical levels during evaluation without fitting on the test data. Numerical predictors were retained as continuous variables. This training-only preprocessing strategy prevented information from the evaluation partition from entering the feature-transformation procedure.

In addition to the fixed hold-out evaluation, formulation-level robustness was assessed using five-fold GroupKFold cross-validation grouped by NM.ID, ensuring zero nanoparticle-identifier overlap between training and validation folds. To quantify variability arising from different formulation partitions, 30 repeated group-aware train–test splits were also evaluated. Performance distributions were summarized using the mean, standard deviation, median, range, and 95% confidence intervals. As a stricter sensitivity analysis, five-fold cross-validation grouped by publication reference was additionally performed to examine transportability to completely unseen studies.

### 2.4 Feature engineering

Feature engineering was performed to extract biologically meaningful and computationally usable predictors from the original database. Particle size was extracted from reported size measurements and represented as a numerical feature. Tumor size was converted into a numerical representation when reported in volume-based or range-based formats. Nanoparticle administration dose was extracted from administration descriptions and converted into a numerical dose variable. Categorical variables were encoded to allow tree-based models to process non-numeric experimental descriptors.

The final feature matrix X contained ten predictor variables (Equation 1):

$$X=\left\{x_{1},x_{2},\ldots ,x_{10}\right\}$$ (1)

where the selected predictors included core material, shape, tumor cell type, animal model, particle size, tumor size, dose, PDI, zeta potential, and time point. The target vector y represented tumor biodistribution efficiency measured as %ID. This feature set was selected because these variables represent key physicochemical and biological factors that can influence nanoparticle transport, circulation, tumor accumulation, and biodistribution behavior.

### 2.5 Machine learning models

Machine learning techniques were employed to discern nonlinear correlations between nanoparticle attributes and tumor delivery efficacy. Tree-based and ensemble learning approaches were chosen due to their suitability for structured biomedical datasets comprising both categorical and numerical information. These models have been extensively employed in predictive modelling tasks and can encapsulate intricate interactions among input variables (Rodriguez-Galiano et al., 2015; Bishop, 2006). The assessed models comprised Random Forest, XGBoost, LightGBM, weighted ensemble learning, and stacked ensemble learning.

#### 2.5.1 Random Forest

Random Forest is an ensemble learning technique that constructs many decision trees and aggregates their predictions. Each tree is constructed using a bootstrap sample of the training data, with random subsets of features being evaluated during the process. In regression, the ultimate prediction is the mean of the predictions from all individual trees (Equation 2):

$${\hat{y}}_{RF}=\frac{1}{N}\overset{N}{\underset{i=1}{\sum }}T_{i}\left(x\right)$$ (2)

where $T_{i}\left(x\right)$ is the prediction of the $i^{th}$ decision tree and N is the total number of trees. Random Forest was included as a strong baseline model due to its robustness, ability to model non-linear relationships, and built-in feature importance estimation.

#### 2.5.2 XGBoost

Extreme Gradient Boosting (XGBoost) is a gradient boosting algorithm that builds trees sequentially, where each new tree attempts to correct the residual errors of the previous trees. The final prediction is represented as an additive ensemble (Equation 3):

$${\hat{y}}_{\mathrm{XGB}}=\overset{K}{\underset{k=1}{\sum }}f_{k}\left(x\right)$$ (3)

where $f_{k}\left(x\right)$ denotes the prediction function of the $k^{th}$ regression tree and K is the number of boosting iterations. XGBoost was selected because gradient boosting models are effective for structured tabular data and can capture nonlinear effects and feature interactions.

#### 2.5.3 LightGBM

Light Gradient Boosting Machine (LightGBM) is another gradient boosting framework designed for efficient training on structured datasets. It uses histogram-based decision tree learning and leaf-wise tree growth to improve computational efficiency. LightGBM was included as a comparative boosting model to evaluate whether a more computationally efficient gradient boosting approach could improve nanoparticle biodistribution prediction.

#### 2.5.4 Weighted ensemble

A weighted ensemble was constructed from the predictions of Random Forest, XGBoost, and LightGBM. To prevent information from the final hold-out test set from influencing ensemble construction, the ensemble weights were estimated exclusively from out-of-fold (OOF) predictions generated within the training partition. Five-fold GroupKFold cross-validation grouped by NM.ID was used to generate one OOF prediction from each base learner for every training record. Preprocessing and model fitting were performed independently within each fold.

The weighted ensemble prediction was defined as Equation 4:

$${\hat{y}}_{WE}=\overset{M}{\underset{i=1}{\sum }}w_{i}{\hat{y}}_{i}$$ (4)

where ${\hat{y}}_{i}$ denotes the prediction of the $i^{th}$ base model, $w_{i}$ is its corresponding weight, and M is the number of base learners. The weights were optimized on the training-only OOF predictions by minimizing RMSE subject to non-negative weights and the constraint $\sum_{i=1}^{M}w_{i}=1$ . The resulting weights were 0.3049 for Random Forest, 0.3433 for XGBoost, and 0.3518 for LightGBM. These weights were fixed before evaluating the final hold-out test set.

#### 2.5.5 Stacking ensemble

A stacking ensemble was also evaluated to determine whether a learned combination of the three base learners could improve predictive performance. Ensemble approaches can exploit complementary predictive information from multiple learning algorithms (Mahdi et al., 2025b). The same training-only NM.ID-grouped OOF predictions from Random Forest, XGBoost, and LightGBM were used as three meta-features. A Ridge regression model with α=1.0 was fitted to these OOF predictions as the second-level meta-model. The fitted stacking model had an intercept of 0.5666 and coefficients of −0.0139 , 0.4309, and 0.4076 for Random Forest, XGBoost, and LightGBM, respectively.

After the ensemble parameters had been determined, the tuned base-model pipelines were fitted on the complete training partition. Their predictions for the untouched hold-out set were then combined using the fixed weighted-ensemble coefficients or supplied to the fixed Ridge meta-model. Consequently, neither the weighted-ensemble optimization nor the stacking meta-model used the final hold-out outcomes during model construction.

#### 2.5.6 Group-aware hyperparameter optimization

Optimization methods, including stochastic and metaheuristic approaches such as the Firefly Algorithm, have been widely applied to machine-learning hyperparameter search (Yang, 2009; 2010). In the revised analysis, hyperparameter optimization was performed separately for Random Forest, XGBoost, and LightGBM using randomized search restricted to the training partition. To preserve formulation-level independence during model selection, five-fold GroupKFold cross-validation was used with NM.ID as the grouping variable. Each candidate configuration was evaluated using root mean squared error (RMSE), and 20 randomly sampled configurations were assessed for each model using a fixed random seed of 42. Categorical encoding was incorporated within the model-selection pipeline so that the one-hot encoder was fitted independently to the training portion of each cross-validation fold.

For Random Forest, the search space included 200, 300, or 500 trees; maximum tree depths of 6, 10, 15, or unrestricted depth; minimum samples per split of 2, 5, or 10; minimum samples per leaf of 1, 2, or 4; and maximum-feature settings of 1.0, 0.7, or the square-root transformation. The selected configuration used 500 trees, a maximum depth of 15, a minimum of 10 samples per split, a minimum of four samples per leaf, and square-root feature sampling.

For XGBoost, the search considered 200, 300, or 500 estimators; maximum depths of 3, 4, 6, or 8; learning rates of 0.02, 0.05, or 0.10; subsampling ratios of 0.7, 0.8, or 1.0; column-sampling ratios of 0.7, 0.8, or 1.0; and minimum child weights of 1, 3, or 5. The selected configuration comprised 300 estimators, a maximum depth of 6, a learning rate of 0.02, a subsampling ratio of 0.7, a column-sampling ratio of 1.0, and a minimum child weight of 3.

For LightGBM, the search space included 200, 300, or 500 estimators; learning rates of 0.02, 0.05, or 0.10; maximum depths of 4, 6, 10, or unrestricted depth; 15, 31, or 63 leaves; minimum child-sample values of 10, 20, or 40; subsampling ratios of 0.7, 0.8, or 1.0; and column-sampling ratios of 0.7, 0.8, or 1.0. The selected configuration used 300 estimators, a maximum depth of 4, 63 leaves, a learning rate of 0.05, 20 minimum child samples, a subsampling ratio of 0.7, and a column-sampling ratio of 1.0. All hyperparameter selection was completed without access to the final 97-NM.ID hold-out test partition.

### 2.6 Model evaluation metrics

Model performance was evaluated using mean absolute error MAE , root mean squared error RMSE , and the coefficient of determination $R^{2}$ . These metrics were selected because the prediction task was formulated as a regression problem.

The MAE was calculated as Equation 5:

$$MAE=\frac{1}{n}\overset{n}{\underset{i=1}{\sum }}|y_{i}-{\hat{y}}_{i}|$$ (5)

where $y_{i}$ is the observed biodistribution value, ${\hat{y}}_{i}$ is the predicted value, and n is the number of samples.

The RMSE was computed as Equation 6:

$$RMSE=\sqrt{\frac{1}{n}\overset{n}{\underset{i=1}{\sum }}{\left(y_{i}-{\hat{y}}_{i}\right)}^{2}}$$ (6)

The coefficient of determination was calculated as Equation 7:

$$R^{2}=1-\frac{\sum_{i=1}^{n}{\left(y_{i}-{\hat{y}}_{i}\right)}^{2}}{\sum_{i=1}^{n}{\left(y_{i}-\bar{y}\right)}^{2}}$$ (7)

where $\bar{y}$ represents the mean of the observed biodistribution values. Higher $R^{2}$ values indicate stronger predictive performance, while lower MAE and RMSE values indicate smaller prediction errors.

In addition to evaluation on the untouched hold-out test set, model robustness was assessed using group-aware validation strategies designed to prevent repeated nanoparticle identities from appearing across training and validation partitions. First, five-fold GroupKFold cross-validation was performed using NM.ID as the grouping variable, ensuring that all records associated with the same nanoparticle identifier remained within a single fold. Performance was summarized across the five folds using MAE, RMSE, and $R^{2}$ , together with 95% confidence intervals for the mean $R^{2}$ .

To further assess sensitivity to a particular train–test partition, 30 repeated group-aware train–test evaluations were conducted using GroupShuffleSplit with NM.ID as the grouping variable and a test fraction of 20%. Random seeds from 0 to 29 were used to generate the 30 partitions. For each model, the resulting performance distribution was summarized using the mean, standard deviation, median, range, and 95% confidence interval.

Finally, a stricter sensitivity analysis was performed using five-fold GroupKFold with the literature Reference field as the grouping variable. This analysis prevented records originating from the same source reference from being distributed across training and validation folds and was used to examine cross-study transportability under a more stringent validation setting.

### 2.7 Explainable artificial intelligence using SHAP

To improve model interpretability, SHapley Additive exPlanations (SHAP) analysis was applied to the fitted XGBoost model as a representative tree-based learner. XGBoost was selected for the explainability analysis because its tree-based structure permits direct application of SHAP-based feature attribution and because it remained one of the competitive base learners under the group-aware cross-validation analysis; this selection should not be interpreted as indicating that XGBoost was consistently superior to the other evaluated models. SHAP assigns each feature a contribution value describing its contribution to an individual model prediction, thereby enabling both global and local interpretation of the predictive relationships learned by the fitted XGBoost model.

For a prediction function f(x) , the SHAP explanation can be expressed as Equation 8:

$$f\left(x\right)=\phi_{0}+\overset{p}{\underset{j=1}{\sum }}\phi_{j},$$ (8)

where $\phi_{0}$ is the baseline prediction, $\phi_{j}$ is the SHAP value for feature j , and p is the number of input features.

Global feature importance was computed using the mean absolute SHAP value (Equation 9):

$$I_{j}=\frac{1}{n}\overset{n}{\underset{i=1}{\sum }}|\phi_{ij}|,$$ (9)

where $I_{j}$ represents the global importance of feature j , and $\phi_{ij}$ is the SHAP value of feature j for sample i . SHAP summary and bar plots were generated to identify the most influential nanoparticle and biological factors associated with tumor biodistribution prediction.

## 3 Results and discussion

### 3.1 Comparative performance of machine learning models

The predictive performance of the developed models was evaluated on the untouched NM.ID-grouped hold-out test set comprising 447 records from 97 nanoparticle identifiers, with no NM.ID overlap with the training partition. Model performance was quantified using mean absolute error (MAE), root mean squared error (RMSE), and the coefficient of determination $\left(R^{2}\right)$ . The resulting hold-out performance is summarized in Table 2.

**TABLE 2:** Performance on the untouched NM.ID-grouped hold-out test set comprising 447 records from 97 nanoparticle identifiers.

Model	MAE	RMSE	$R^{2}$
Stacking ensemble	1.9291	3.8107	0.0901
Weighted ensemble	1.8320	3.8132	0.0889
Random forest	1.9230	3.8149	0.0881
LightGBM	1.8841	3.8416	0.0752
XGBoost	1.8612	3.9271	0.0336

Table 2 shows that the differences among the evaluated models were relatively small under the more stringent NM.ID-grouped hold-out design. The stacking ensemble achieved the lowest RMSE (3.8107) and the highest $R^{2}$ (0.0901), whereas the weighted ensemble achieved the lowest MAE (1.8320). Random Forest produced nearly comparable performance, with an RMSE of 3.8149 and an $R^{2}$ of 0.0881. LightGBM and XGBoost achieved $R^{2}$ values of 0.0752 and 0.0336, respectively. These findings indicate that no individual learning algorithm demonstrated a substantial predictive advantage under evaluation on previously unseen nanoparticle identities.

The weighted and stacking ensemble approaches provided only marginal improvements over the strongest individual model. Relative to Random Forest, the weighted ensemble reduced RMSE by only 0.0017 and increased $R^{2}$ by 0.0008, while the stacking ensemble reduced RMSE by 0.0042 and increased $R^{2}$ by 0.0020. Importantly, the ensemble parameters were determined exclusively from training-set out-of-fold predictions and were fixed before evaluation of the final hold-out set. Thus, the revised ensemble analysis avoids using hold-out outcomes for model construction and provides a more independent assessment of ensemble performance.

Although XGBoost did not outperform the other approaches under the revised formulation-grouped hold-out evaluation, gradient boosting remains well suited to modelling nonlinear relationships among nanoparticle physicochemical properties, tumor characteristics, and experimental factors. Previous studies have similarly demonstrated the utility of gradient boosting and related machine-learning approaches for nanoparticle biodistribution prediction and nanomedicine optimization (Mahdi et al., 2025a; Ma et al., 2023; Bannigan et al., 2021). Such methods can capture nonlinear interactions among physicochemical and biological variables that may influence nanoparticle accumulation and tumor delivery efficiency (Albanese et al., 2012; Banerjee et al., 2016; Xu et al., 2023).

More broadly, the present findings remain consistent with growing evidence that machine-learning models can provide useful computational support for studying nanoparticle transport and assisting nanomedicine design (Lin et al., 2022; Mi et al., 2024; Chou and Lin, 2023; Singh et al., 2023). Because conventional experimental screening is resource intensive and frequently involves animal studies, data-driven models may serve as complementary tools for prioritizing candidate formulations and generating hypotheses for subsequent experimental evaluation (Burden et al., 2017; Poon et al., 2020). However, the comparatively modest performance observed under formulation-grouped validation emphasizes that such models should complement rather than replace experimental validation.

The predictive patterns considered in this study further support the relevance of physicochemical and experimental characteristics such as particle size, surface charge, material composition, and administration conditions. Previous investigations have demonstrated that these factors can influence nanoparticle biodistribution and tumor-targeting behavior (Perrault et al., 2009; Wilhelm et al., 2016; Ikeda-Imafuku et al., 2022; Li et al., 2022; 2020). Nevertheless, associations learned by the present machine-learning models should not be interpreted as causal relationships, and their predictive relevance depends on the domain represented by the training data.

Overall, the revised hold-out evaluation demonstrates that generalization to nanoparticle identities not encountered during training is substantially more challenging than suggested by the original row-wise evaluation. The stacking ensemble achieved the numerically highest hold-out $R^{2}$ (0.0901), but its advantage over Random Forest and the weighted ensemble was minimal. Therefore, the revised results do not support designating a single algorithm as clearly superior. Instead, they provide a more conservative estimate of generalization performance and highlight both the potential and the current limitations of explainable machine-learning approaches for supporting nanoparticle design and cancer nanomedicine research.

### 3.2 Cross-validation analysis

Because performance from a single hold-out partition may depend on the particular nanoparticle identities assigned to each subset, five-fold group-aware cross-validation was additionally performed using NM.ID as the grouping variable. All records belonging to the same nanoparticle identifier were retained within the same fold, preventing identity-level overlap between training and validation data. Mean MAE, RMSE, and $R^{2}$ across the five folds are summarized in Table 3, together with the 95% confidence interval for the mean $R^{2}$ .

**TABLE 3:** Five-fold NM.ID-grouped cross-validation performance of the evaluated base machine learning models.

Model	MAE mean	RMSE mean	$R^{2}$ mean	95% CI for $R^{2}$
XGBoost	1.9259	3.8689	0.2280	[0.0011, 0.4549]
Random forest	2.0504	3.9183	0.2169	[-0.0139, 0.4477]
LightGBM	1.9239	3.9373	0.1980	[-0.0246, 0.4207]

Under five-fold NM.ID-grouped cross-validation, the three base models produced substantially more conservative and closely clustered performance estimates than those obtained under the original row-wise validation design. XGBoost achieved the numerically highest mean $R^{2}$ (0.2280; 95% CI: 0.0011–0.4549), followed by Random Forest ( $R^{2}=0.2169$ ; 95% CI: −0.0139 –0.4477) and LightGBM ( $R^{2}=0.1980$ ; 95% CI: −0.0246 –0.4207). LightGBM produced the lowest mean MAE (1.9239), whereas XGBoost produced the lowest mean RMSE (3.8689). The overlapping confidence intervals and small differences among the mean performance estimates do not support a clear superiority claim for any single base learner.

The reduction in cross-validation performance relative to the original row-wise analysis is consistent with the presence of repeated measurements and heterogeneous nanoparticle formulations and biological conditions in the database. When records associated with the same nanoparticle identity are prevented from occurring across training and validation folds, the evaluation more directly assesses generalization to previously unseen nanoparticle identities. Heterogeneity in physicochemical properties, tumor microenvironments, pharmacokinetic conditions, and experimental procedures has also been recognized as an important source of variability in nanoparticle delivery studies (Cheng et al., 2020; Dogra et al., 2020; Price et al., 2020).

Although gradient-boosting and ensemble-tree methods are capable of modelling complex nonlinear interactions between nanoparticle characteristics and biological factors, their performance remains dependent on the representativeness of the training data. Previous studies have demonstrated the usefulness of machine-learning and ensemble approaches for biomedical and nanomedicine prediction problems (Bannigan et al., 2021; Chou and Lin, 2023; Singh et al., 2023), while computational modelling has increasingly contributed to nanoparticle pharmacokinetic and delivery research (Dogra et al., 2020; Chou and Lin, 2023). The present group-aware results indicate, however, that algorithmic flexibility alone does not eliminate the difficulty of generalizing across distinct nanoparticle formulations.

These findings also explain the apparent discrepancy between the comparatively high performance obtained under the original row-wise cross-validation and the more modest performance observed under formulation-grouped evaluation. The original random partitioning allowed measurements associated with the same or closely repeated nanoparticle identities to occur across training and validation subsets, producing an optimistic estimate of generalization. In contrast, the revised NM.ID-grouped analysis removes this identity-level overlap and therefore provides a more stringent assessment of performance on unseen nanoparticle identities.

Overall, the group-aware cross-validation results demonstrate substantial variability in generalization across nanoparticle identities and do not establish a universally superior model. Rather than supporting claims of uniformly high predictive accuracy, the revised analysis provides a more conservative assessment of the current framework and emphasizes the importance of identity-aware validation when machine-learning models are evaluated for nanoparticle screening and formulation research.

#### 3.2.1 Repeated group-aware validation and cross-study sensitivity

To assess whether the results depended strongly on a particular group-aware partition, 30 additional train–test evaluations were performed using GroupShuffleSplit, with NM.ID as the grouping variable, a test fraction of 20%, and random seeds ranging from 0 to 29. Table 4 summarizes the resulting performance distributions. Across the 30 splits, Random Forest achieved a mean $R^{2}$ of 0.1419 (SD = 0.1299; 95% CI: 0.0934–0.1904), XGBoost achieved a mean $R^{2}$ of 0.1055 (SD = 0.1591; 95% CI: 0.0462–0.1649), and LightGBM achieved a mean $R^{2}$ of 0.0924 (SD = 0.1715; 95% CI: 0.0284–0.1565). The corresponding mean MAE values were 2.1191, 2.0540, and 2.0694, respectively.

**TABLE 4:** Performance across 30 repeated NM.ID-grouped train–test splits. Values are reported as mean ± standard deviation, with 95% confidence intervals for the mean.

Model	MAE mean ± SD	MAE 95% CI	$R^{2}$ mean ± SD	$R^{2}$ 95% CI
Random forest	2.1191 ± 0.4273	[1.9596, 2.2787]	0.1419 ± 0.1299	[0.0934, 0.1904]
XGBoost	2.0540 ± 0.4346	[1.8917, 2.2162]	0.1055 ± 0.1591	[0.0462, 0.1649]
LightGBM	2.0694 ± 0.4535	[1.9001, 2.2388]	0.0924 ± 0.1715	[0.0284, 0.1565]

The repeated-split analysis showed substantial variability across different assignments of nanoparticle identities to the training and test partitions. Although Random Forest produced the numerically highest mean $R^{2}$ , the overlapping performance distributions and variation across splits indicate that model ranking was sensitive to the particular nanoparticle identities included in each partition. These results therefore reinforce the conclusion from the five-fold group-aware analysis that no evaluated base learner demonstrated a consistent and substantial advantage across formulation-grouped partitions.

As an additional and more stringent sensitivity analysis, five-fold GroupKFold validation was performed using the literature Reference field as the grouping variable. This design prevented records originating from the same source reference from occurring in both training and validation folds. Under this cross-study grouping, mean $R^{2}$ values decreased to −0.1579 for Random Forest, −0.4270 for LightGBM, and −0.4491 for XGBoost. Negative $R^{2}$ values indicate that, under this particularly stringent cross-study setting, the models did not outperform a mean-response baseline on average.

The deterioration under reference-grouped validation should not be interpreted as invalidating the within-database modelling results. Rather, it indicates that substantial between-study heterogeneity remains in the compiled database and that generalization across entirely separated literature sources is considerably more difficult than interpolation within the represented experimental domain. Differences in nanoparticle characteristics, tumor models, experimental protocols, pharmacokinetic conditions, and measurement procedures may contribute to this cross-study variability (Cheng et al., 2020; Dogra et al., 2020; Price et al., 2020). Consequently, external validation on independently collected datasets remains necessary before the framework can be considered reliable for prospective prediction across new experimental studies.

#### 3.2.2 Dose–target relationship and ablation analysis

\Because the prediction target was tumor biodistribution expressed as percentage injected dose (%ID), the relationship between administered dose and the target was examined explicitly. Dose showed essentially no marginal linear or monotonic association with tumor %ID. Pearson correlation analysis yielded r=−0.0141 (p=0.498) , while Spearman rank correlation yielded ρ=0.0064 (p=0.757) . Thus, variation in administered dose was not directly associated with variation in tumor %ID in the analyzed dataset.

To further evaluate whether dose contributed useful predictive information in the multivariable machine-learning framework, an ablation analysis was performed using the same five-fold NM.ID-grouped cross-validation procedure. Each base model was evaluated with the complete predictor set and again after excluding Dose_num. The results are summarized in Table 5.

**TABLE 5:** Dose ablation analysis under five-fold NM.ID-grouped cross-validation.

Model	Predictors	MAE	RMSE	$R^{2}$
XGBoost	With dose	1.9259	3.8689	0.2280
	Without dose	1.9463	3.9143	0.2108
Random forest	With dose	2.0504	3.9183	0.2169
	Without dose	2.0837	3.9550	0.2007
LightGBM	With dose	1.9239	3.9373	0.1980
	Without dose	2.0281	4.0993	0.1236

Removing dose did not improve predictive performance for any of the three base models. The mean $R^{2}$ decreased from 0.2280 to 0.2108 for XGBoost, from 0.2169 to 0.2007 for Random Forest, and from 0.1980 to 0.1236 for LightGBM. Corresponding increases in MAE and RMSE were also observed. These results indicate that, despite the absence of a meaningful marginal correlation with tumor %ID, dose provides modest contextual predictive information when considered jointly with the other physicochemical, biological, and experimental variables. This finding does not imply an independent or causal effect of administered dose on tumor %ID.

#### 3.2.3 Generalization to unseen core materials

To further examine generalization beyond categorical levels represented during model training, performance was evaluated separately for test records containing core-material categories that were either present or absent in the training partition. In the final NM.ID-grouped hold-out set, 394 of 447 records contained core-material categories represented during training, whereas 53 records (11.86%) belonged to 11 core-material categories that were completely absent from the training partition.

The unseen core-material categories were Iridium, Other-Au@C60, Other-CaP, Other-Cobalt, Other-HCPT NR, Other-PMCN, Platinum, Polymeric/Liposome, Ruthenium, Silica/Polymeric, and Silver. Table 6 compares predictive performance between records with seen and unseen core-material categories.

**TABLE 6:** Predictive performance on seen and unseen core-material categories in the final NM.ID-grouped hold-out set.

Model	Core material	MAE	RMSE	$R^{2}$
Random forest	Seen	1.8222	3.6342	0.0879
	Unseen	2.6727	4.9556	0.0773
XGBoost	Seen	1.7498	3.7093	0.0498
	Unseen	2.6896	5.2712	−0.0440
LightGBM	Seen	1.7997	3.7110	0.0490
	Unseen	2.5116	4.7005	0.1698
Weighted ensemble	Seen	1.7299	3.6325	0.0888
	Unseen	2.5908	4.9543	0.0778
Stacking ensemble	Seen	1.8212	3.6266	0.0917
	Unseen	2.7316	4.9700	0.0719

Prediction errors were generally higher for records containing unseen core-material categories. For example, the weighted ensemble MAE increased from 1.7299 for seen materials to 2.5908 for unseen materials, while RMSE increased from 3.6325 to 4.9543. XGBoost showed a similar deterioration, with MAE increasing from 1.7498 to 2.6896 and RMSE from 3.7093 to 5.2712. Random Forest and the stacking ensemble exhibited comparable increases in prediction error.

Although LightGBM yielded a positive $R^{2}$ of 0.1698 for the unseen-material subset, this result was based on only 53 records distributed across 11 heterogeneous unseen categories and should not be interpreted as evidence of reliable extrapolation to arbitrary new nanoparticle chemistries. Across the models, the generally increased MAE and RMSE indicate that prediction becomes more difficult when the core-material category is not represented during training.

These results demonstrate an important distinction between technical handling of unseen categorical levels and validated chemical extrapolation. The one-hot encoding procedure with unknown-category handling allows the trained pipelines to generate predictions without an encoding error when an unseen categorical level is encountered. However, the present results do not establish reliable prediction for completely novel nanoparticle materials. Accordingly, the proposed framework should primarily be regarded as an interpolation and screening approach within the chemical and experimental domain represented by the training data. Prospective prediction of genuinely novel nanoparticle chemistries will require independent external validation and may benefit from chemically informative representations such as molecular descriptors, fingerprints, or learned material embeddings.

### 3.3 SHAP-based explainability analysis

In addition to evaluating predictive performance, model interpretability was examined using SHapley Additive exPlanations (SHAP). SHAP values were used to characterize how individual predictors contributed to the model outputs and to provide global and local descriptions of the learned predictive relationships. Because the revised group-aware evaluation did not identify a single model with a substantial and consistent performance advantage, the SHAP results are interpreted as descriptions of associations learned by the fitted model rather than as evidence of causal or mechanistic effects.

Figure 2 presents the SHAP summary plot, while Table 7 and Figure 3 summarize global feature importance for the final tuned XGBoost model. After aggregating SHAP contributions from one-hot encoded categorical levels to the corresponding original predictors, Animal showed the highest mean absolute SHAP value (0.4762), followed by Tumor Cell (0.4272), Core material (0.3080), Dose_num (0.2955), and Zeta Potential (0.2680). Time point (h), Size_num, PDI, Shape, and TumorSize_num showed progressively smaller global contributions. These SHAP values describe predictive relationships learned by the fitted model and should not be interpreted as evidence of causal effects on nanoparticle tumor accumulation.

**FIGURE 2:**
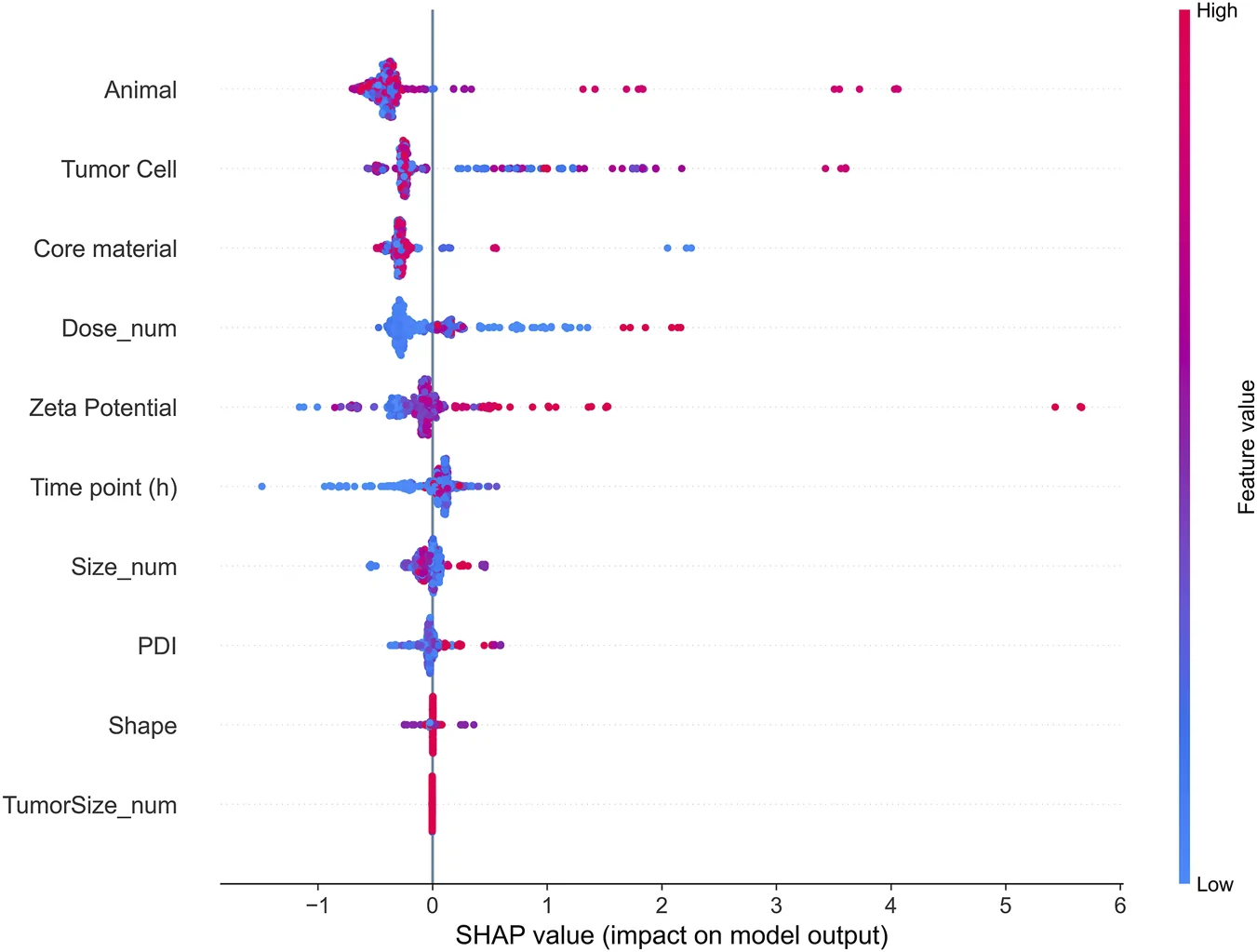
SHAP summary plot for the final tuned XGBoost model evaluated on the untouched NM.ID-grouped hold-out set. SHAP values for one-hot encoded categorical levels were aggregated to the corresponding original predictor. Each point represents one test observation; horizontal position indicates the contribution to the model output, while color represents the feature value used for visualization.

**TABLE 7:** Global feature importance for the final tuned XGBoost model based on mean absolute SHAP values aggregated to the original predictor level.

Rank	Feature	Mean absolute SHAP value
1	Animal	0.4762
2	Tumor cell	0.4272
3	Core material	0.3080
4	Dose_num	0.2955
5	Zeta potential	0.2680
6	Time point (h)	0.1524
7	Size_num	0.0745
8	PDI	0.0595
9	Shape	0.0171
10	TumorSize_num	0.0025

**FIGURE 3:**
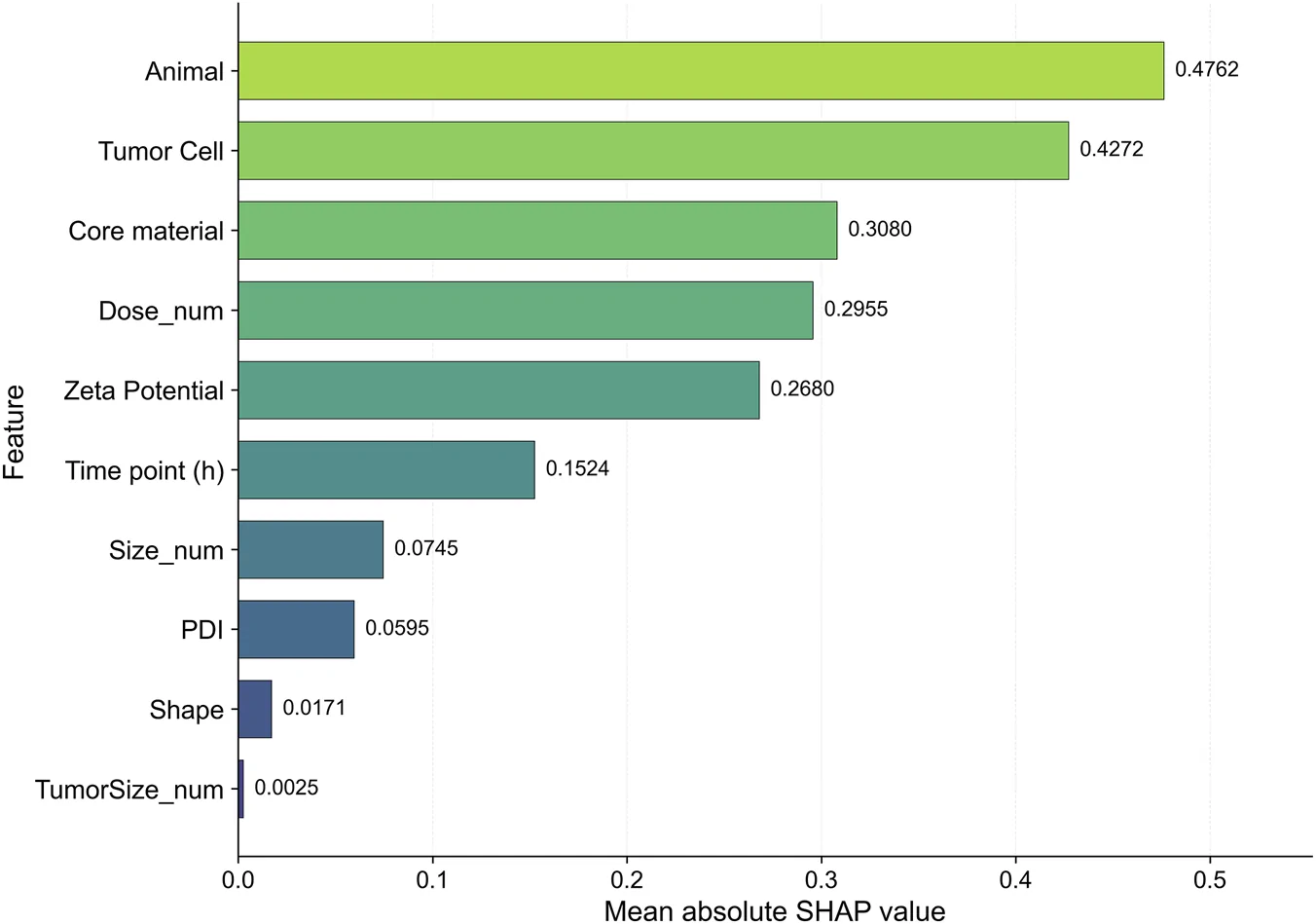
Global feature importance for the final tuned XGBoost model based on mean absolute SHAP values aggregated to the original predictor level. Animal and Tumor Cell showed the largest mean absolute SHAP values, followed by Core material, Dose_num, and Zeta Potential.

Although tumor-related characteristics are biologically relevant to nanoparticle transport and retention, TumorSize_num showed only a small contribution in the revised SHAP analysis. This finding does not imply that tumor size is biologically unimportant; rather, it indicates that this variable contributed little to predictions from the fitted XGBoost model after leakage-resistant preprocessing and group-aware model development. Tumor vascularization, interstitial pressure, tissue architecture, and other microenvironmental characteristics can influence nanoparticle delivery and the enhanced permeability and retention (EPR) effect (Shi et al., 2017; Ikeda-Imafuku et al., 2022; Cheng et al., 2020). Physiologically based pharmacokinetic studies have likewise demonstrated the relevance of tumor-specific factors to nanoparticle biodistribution (Cheng et al., 2020; Dogra et al., 2020). The low SHAP importance of TumorSize_num in the present model should therefore be interpreted as a model-specific predictive result rather than evidence against the broader biological importance of the tumor microenvironment.

Zeta Potential remained among the more influential physicochemical predictors in the revised SHAP analysis, ranking fifth by mean absolute SHAP value. Surface charge is known to affect protein corona formation, cellular uptake, blood circulation time, and interactions with biological membranes, and nanoparticles with different surface charges may exhibit different biodistribution and clearance profiles (Albanese et al., 2012; Tang et al., 2019; Li et al., 2020). Previous studies have also shown that modification of nanoparticle surface properties can affect tumor penetration and therapeutic performance (Li et al., 2022; Perrault et al., 2009). In the present analysis, however, the SHAP contribution of Zeta Potential should be interpreted as a model-specific predictive association rather than evidence of a direct causal effect on tumor delivery efficiency.

Dose_num requires careful interpretation because the prediction target is tumor biodistribution expressed as percentage injected dose (%ID), which is normalized to the administered amount. Therefore, dose and %ID should not be interpreted as algebraically equivalent quantities, and the present analysis does not imply a direct causal relationship between administered dose and tumor delivery efficiency. Consistent with this distinction, dose showed essentially no marginal association with the target (Pearson r=−0.0141 , p=0.498 ; Spearman ρ=0.0064 , p=0.757 ).

Nevertheless, dose was retained as a predictor because administered amount may contain contextual information related to experimental conditions and may participate in nonlinear interactions with other physicochemical and biological variables. This interpretation is consistent with pharmacokinetic studies in which administration conditions are considered alongside nanoparticle and biological characteristics (Price et al., 2020; Dogra et al., 2020). A group-aware ablation analysis further showed that removing dose did not improve predictive performance. Under five-fold NM.ID-grouped cross-validation, the mean $R^{2}$ decreased from 0.2280 to 0.2108 for XGBoost, from 0.2169 to 0.2007 for Random Forest, and from 0.1980 to 0.1236 for LightGBM when dose was excluded. Thus, dose was retained as a contextual predictive feature, while its model importance should not be interpreted as evidence that dose independently or causally determines tumor %ID.

Core material ranked third in the revised global SHAP analysis, whereas Size_num and Shape showed smaller global contributions, ranking seventh and ninth, respectively. These model-specific differences do not imply that particle size or shape is biologically unimportant. Experimental literature indicates that particle composition, shape, and size can affect circulation dynamics, immune recognition, tissue penetration, and cellular uptake (Xu et al., 2023; Banerjee et al., 2016; Albanese et al., 2012), and studies have shown that nanoparticle size optimization can influence tumor accumulation and off-target distribution (Poon et al., 2020; Perrault et al., 2009; Xu et al., 2023). The present SHAP ranking should therefore be interpreted as describing the relative contributions of these variables within the fitted dataset and model rather than as a universal hierarchy of nanomedicine design factors.

The interpretability findings are broadly consistent with previous applications of explainable artificial intelligence to nanoparticle delivery prediction. Using an XGBoost-SHAP framework, Ma et al. similarly identified nanoparticle physicochemical properties as important contributors to model predictions of delivery efficiency (Ma et al., 2023). Machine-learning-guided analyses have increasingly been used to identify potentially relevant design characteristics in nanomedicine research (Bannigan et al., 2021; Singh et al., 2023; Chou and Lin, 2023). In the present study, SHAP improves transparency by showing which variables contributed most strongly to the fitted model outputs; however, these feature attributions should be interpreted as model-specific predictive associations and not as causal evidence or independently validated design rules.

Overall, the SHAP analysis indicates that tumor characteristics, nanoparticle surface properties, administration conditions, and material composition contribute to the predictive patterns learned by the model. These results provide an interpretable description of the fitted model and generate biologically plausible hypotheses for further investigation, but they should not be regarded as demonstrating mechanisms or causal determinants of nanoparticle tumor delivery. Experimental and external validation will be required before these model-derived associations can be translated into nanoparticle design recommendations.

### 3.4 Biological interpretation and comparison with previous studies

The results obtained in this work demonstrate both the potential and the limitations of machine learning for describing associations among nanoparticle physicochemical properties, tumor characteristics, experimental conditions, and delivery efficiency. The group-aware evaluation showed that predictive generalization to unseen nanoparticle identities is considerably more difficult than suggested by row-wise validation, while SHAP provides a transparent description of the predictive relationships learned by the fitted model. Accordingly, the interpretability results are considered hypothesis-generating rather than mechanistic evidence for nanoparticle design.

A critical observation is the predominant impact of tumor-specific attributes on the efficacy of nanoparticle administration. Animal model and tumor cell type were identified as the two most influential predictors in the SHAP analysis, while TumorSize_num showed only a small contribution. These findings indicate that biological context contributes substantially to the predictive patterns learned by the model. Prior studies have similarly shown that vascular permeability, tumor heterogeneity, and interstitial fluid pressure substantially affect nanoparticle transit and retention in tumors (Shi et al., 2017; Wilhelm et al., 2016; Cheng et al., 2020). These biological barriers continue to pose significant hurdles for enhancing therapeutic delivery in cancer nanomedicine.

The importance of nanoparticle size identified in this study is consistent with numerous experimental and computational investigations. Nanoparticle size directly influences circulation time, tissue penetration, cellular uptake, and clearance pathways. Xu et al. reported strong size-dependent effects on nanoparticle transport and biodistribution, demonstrating that optimized particle dimensions can substantially improve targeting efficiency while reducing systemic clearance (Xu et al., 2023). Similar observations were previously reported by Albanese et al. and Banerjee et al., who highlighted nanoparticle size as a key determinant of biological interactions and delivery outcomes (Albanese et al., 2012; Banerjee et al., 2016).

Surface-related properties also played a major role in the predictive framework. In particular, zeta potential was identified as one of the strongest predictors of tumor delivery efficiency. Surface charge affects protein adsorption, immune recognition, blood circulation, and cellular internalization, thereby influencing overall biodistribution behavior (Tang et al., 2019; Li et al., 2020). Recent advances in nanoparticle engineering have focused on modifying surface properties to improve tumor penetration and therapeutic efficacy, further emphasizing the biological relevance of the variables identified by the proposed model (Li et al., 2022; Perrault et al., 2009).

The results are also consistent with large-scale analyses of nanoparticle biodistribution. Chen et al. expanded the earlier Nano-Tumor resource from 376 nanoparticle datasets to 534 tumor datasets comprising 2,345 time-dependent tumor concentration data points from 297 studies (Chen et al., 2023). This expanded resource provides substantially broader coverage of nanoparticle formulations and experimental settings than the earlier database used by Lin et al. (Lin et al., 2022).

Direct numerical comparison with earlier predictive studies requires caution because the modeled endpoints, feature sets, performance metrics, and validation procedures differ. Using the earlier 376-dataset resource, Lin et al. reported adjusted $R^{2}$ values of 0.92, 0.77, 0.77, and 0.76 for $DE_{\max}$ , $DE_{24}$ , $DE_{168}$ , and $DE_{\mathrm{Tlast}}$ , respectively, for their deep neural network models (Lin et al., 2022). Ma et al., using an XGBoost-SHAP framework that additionally incorporated tumor genomic mutation information, reported test-set correlation coefficients of 0.66, 0.75, and 0.54 for maximum delivery efficiency and delivery efficiency at 24 and 168 h, respectively (Ma et al., 2023). These values are not directly comparable with the present group-aware $R^{2}$ estimates because correlation coefficients are not equivalent to $R^{2}$ , the prediction endpoints and feature sets differ, and the present analysis explicitly excludes the same nanoparticle identities from training and evaluation. The lower performance obtained under the present leakage-resistant validation should therefore be interpreted in the context of a more stringent generalization task rather than as a direct indication that one modeling approach is superior or inferior to another.

Recent developments in artificial intelligence have accelerated the application of computational approaches to nanomedicine. Machine-learning methods have increasingly been employed for nanoparticle design optimization, biodistribution prediction, and formulation development (Bannigan et al., 2021; Singh et al., 2023; Chou and Lin, 2023). The present results support the use of explainable machine learning as a complementary analytical approach for identifying predictive patterns in nanomedicine datasets, while also demonstrating that apparent predictive performance depends strongly on how repeated nanoparticle identities and heterogeneous source studies are handled during validation. Thus, interpretability and rigorous assessment of generalization should be considered jointly when evaluating machine-learning approaches for nanoparticle delivery prediction.

These computational developments should also be considered within the broader translational challenges of cancer nanomedicine. Nanocarrier-based delivery systems can improve drug targeting, controlled release, and therapeutic delivery, but their performance remains dependent on complex interactions among carrier properties, biological barriers, and tumor characteristics (Sohail et al., 2021). More recent assessments have emphasized that heterogeneous tumor physiology, limitations of passive targeting, protein-corona formation, toxicity assessment, and regulatory standardization continue to constrain clinical translation, while artificial intelligence may contribute to more systematic nanomedicine design and optimization (Sohail et al., 2026). These considerations further support cautious interpretation of computational predictions and the need for experimental and external validation before model-derived findings are translated into prospective nanomedicine design decisions.

The proposed framework also aligns with emerging efforts to use computational screening to complement experimental nanomedicine research. Machine learning-guided screening strategies may help prioritize nanoparticle formulations for subsequent laboratory evaluation and may thereby contribute to more efficient use of experimental resources (Burden et al., 2017; Poon et al., 2020). Given the limited generalization observed under the stricter group-aware and cross-study validation analyses, however, the present framework should be considered a hypothesis-generating and prioritization tool rather than a replacement for experimental validation or a demonstrated means of accelerating clinical translation.

Furthermore, the application of SHAP-based explainability provides greater transparency than conventional black-box predictive models by showing how individual nanoparticle properties and biological factors contribute to the fitted model outputs. These feature attributions facilitate interpretation of the predictive relationships learned by the model but do not establish biological mechanisms or causal effects. Similar applications of explainable artificial intelligence have been reported in studies combining machine learning with nanoparticle delivery prediction and biological characterization (Ma et al., 2023; Kingston et al., 2019). By integrating predictive modeling with interpretable feature attribution, the proposed framework provides a transparent computational approach for hypothesis generation and nanoparticle screening, while its potential clinical relevance requires further external and prospective validation.

An important limitation concerns extrapolation to nanoparticle materials that are not represented during model training. The one-hot encoding procedure used in the present framework permits previously unseen categorical levels to be processed without producing an encoding error; however, this technical capability should not be interpreted as evidence of reliable chemical extrapolation. In the final NM.ID-grouped hold-out set, 53 of 447 records (11.86%) represented 11 core-material categories that were absent from the training partition. Prediction errors were generally higher for these unseen-material records than for records whose core-material categories were represented during training. For example, the weighted ensemble yielded an MAE of 1.7299 and RMSE of 3.6325 for seen core materials, compared with an MAE of 2.5908 and RMSE of 4.9543 for unseen core materials. Similarly, XGBoost produced an MAE of 1.7498 and RMSE of 3.7093 for seen materials, compared with 2.6896 and 5.2712, respectively, for unseen materials. These results indicate that the current framework should primarily be interpreted as an interpolation and screening tool within the chemical and experimental domain represented by the training data rather than as a validated predictor for completely novel nanoparticle chemistries. Reliable extrapolation to new material classes will require external validation and, potentially, chemically informative representations such as molecular descriptors, fingerprints, or learned material embeddings.

Overall, the results demonstrate that explainable machine learning can provide a useful framework for modeling nanoparticle tumor delivery efficiency and identifying predictive patterns associated with biodistribution. The integration of group-aware predictive modeling, repeated validation, sensitivity analysis, and SHAP-based interpretation provides a transparent approach for hypothesis generation and prioritization of nanoparticle formulations. However, the modest performance under formulation-grouped validation and the limited transportability observed across unseen studies and material categories indicate that external and prospective experimental validation is required before the framework can inform translational or clinical applications.

## 4 Conclusion

This study developed an explainable machine-learning framework for predicting nanoparticle tumor delivery efficiency using physicochemical, biological, and experimental characteristics from the updated Nano-Tumor Database. Random Forest, XGBoost, LightGBM, weighted ensemble, and stacking ensemble approaches were evaluated using a leakage-aware validation strategy in which records were grouped by nanoparticle identity (NM.ID). In the final untouched group-aware hold-out set, the stacking ensemble achieved the numerically lowest RMSE (3.8107) and highest $R^{2}$ (0.0901), whereas the weighted ensemble achieved the lowest MAE (1.8320). However, the differences among the leading models were minimal and do not support designation of a single algorithm as clearly superior.

Five-fold NM.ID-grouped cross-validation yielded mean $R^{2}$ values of 0.2280 for XGBoost, 0.2169 for Random Forest, and 0.1980 for LightGBM. Repeated group-aware train–test splits further demonstrated substantial variability in generalization performance and model ranking. Moreover, the stricter reference-grouped sensitivity analysis produced negative mean $R^{2}$ values, indicating limited transportability across completely separated literature sources. Collectively, these findings show that prediction of previously unseen nanoparticle identities and independent experimental studies is considerably more challenging than suggested by conventional row-wise validation and emphasize the importance of group-aware evaluation in nanoparticle machine-learning studies.

SHAP-based explainability identified Animal, Tumor Cell, Core material, Dose_num, and Zeta Potential as the largest global contributors to predictions from the final tuned XGBoost model, while Time point (h), Size_num, PDI, Shape, and TumorSize_num showed smaller global contributions.

Additional analysis clarified that administered dose showed essentially no marginal correlation with tumor %ID, although its removal modestly reduced predictive performance under group-aware cross-validation, suggesting contextual multivariable information rather than a direct or causal relationship. Evaluation of previously unseen core-material categories further demonstrated generally increased prediction errors compared with materials represented during training. Therefore, the ability of the preprocessing pipeline to technically accommodate an unseen categorical level should not be interpreted as validated extrapolation to completely novel nanoparticle chemistries.

The principal value of the proposed framework is consequently its integration of explainable modelling with leakage-aware validation, training-only hyperparameter optimization and ensemble construction, repeated group-aware evaluation, dose ablation, and explicit assessment of unseen material categories. Within these constraints, the framework may serve as a computational screening and hypothesis-generation tool for investigating relationships among nanoparticle characteristics, biological conditions, and tumor delivery efficiency. It should not, however, be considered a substitute for experimental validation or a validated predictor for arbitrary new nanoparticle formulations.

Future research should prioritize external validation using independently collected datasets, broader representation of nanoparticle chemistries and experimental conditions, and prospective experimental assessment of model-generated hypotheses. Chemically informative representations, including molecular descriptors, fingerprints, or learned material embeddings, may improve generalization to material classes not represented during training. Incorporating additional biological, genomic, and pharmacokinetic information may also improve predictive transportability. Overall, the present findings demonstrate both the potential and the current limitations of explainable machine learning for supporting data-driven nanoparticle screening and cancer nanomedicine research.

## Data Availability

Publicly available datasets were analyzed in this study. This data can be found here: The data analyzed in this study were obtained from the publicly available Nano-Tumor Database. The dataset is available at: https://github.com/UFPBPK/Nano-Tumor-Database-Version2023. Processed data generated during the current study are available from the corresponding author upon reasonable request.
